# Case Report: A complete pathologic response in pancreatic cancer with squamous cell differentiation

**DOI:** 10.3389/fonc.2023.1240405

**Published:** 2023-11-29

**Authors:** Christopher R. Grant, Zhaohui L. Arter, Tran Truc, Fa-Chyi Lee

**Affiliations:** ^1^ Department of Medicine, University of California Irvine Medical Center, Orange, CA, United States; ^2^ Hematology/Oncology, University of California Irvine Medical Center, Orange, CA, United States; ^3^ Department of Pathology, University of California, Irvine, Irvine, CA, United States

**Keywords:** case report, pancreatic cancer, squamous cell carcinoma, chemotherapy, complete pathologic response

## Abstract

Primary pancreatic malignancies are mostly composed of the adenocarcinoma histological subtype. However, squamous cell carcinoma (SCC) accounts for approximately 0.5%–1% of all malignant pancreatic cancers. Because of the rarity of SCC of the pancreas, guideline-directed treatment is lacking, treatment response is difficult to access, and treatment options are poorly defined. Here, we report a case of a 65-year-old man diagnosed with pancreatic carcinoma with dominant squamous cell differentiation, who achieved complete pathologic response (CPR) after treatment with gemcitabine, cisplatin, and nab-paclitaxel every 14 days for six cycles and who continues to lead a high quality of life 7 months later. To our knowledge, this is the first case of CPR in a case of SCC of the pancreas. To highlight the ambiguity and the need for further studies, we also performed a narrative review analyzing recent cases and compared them to our case.

## Introduction

**Figure 1:**
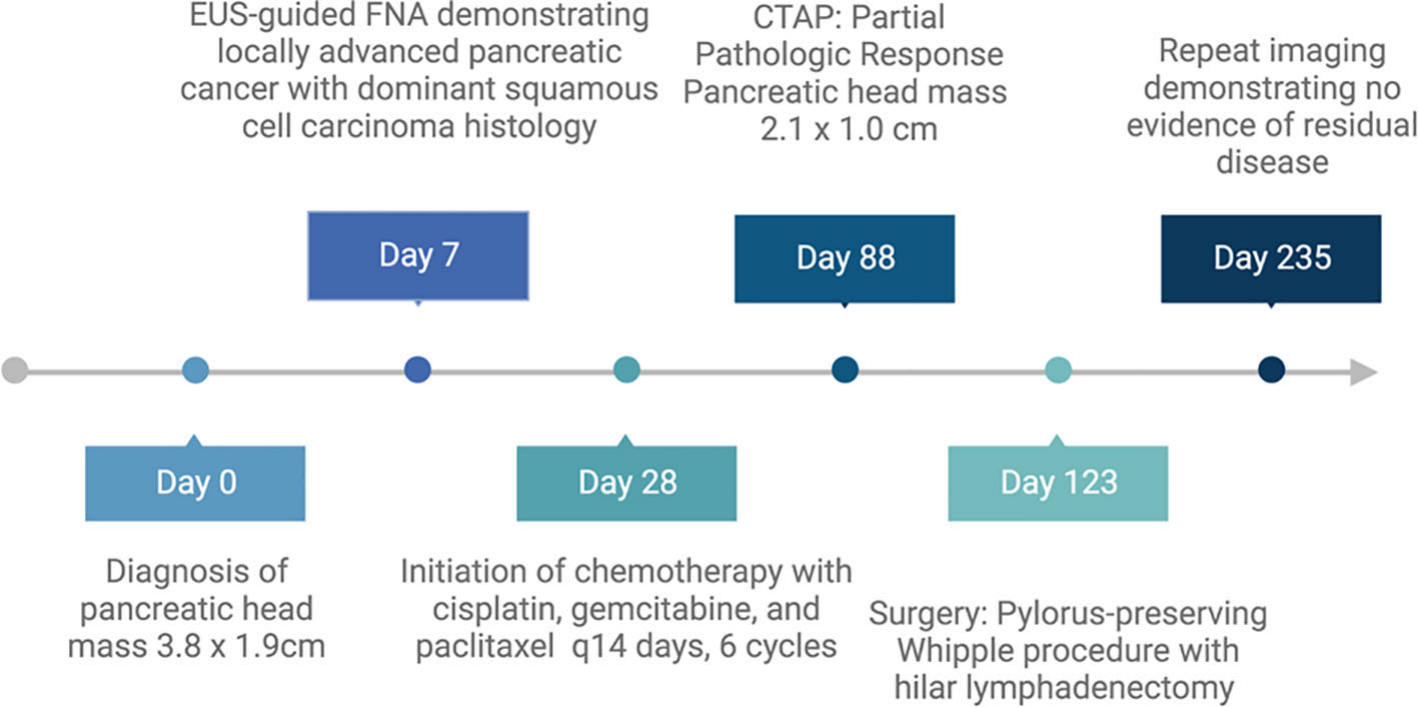
Timeline of patient’s course of treatment. Created with BioRender.com.

Pancreatic carcinoma accounts for about 3% of all cancers in the United States and has an average 5-year relative survival rate of 44% when localized. This rate decreases to 3% when distant metastasis is present (1). Squamous cell carcinoma (SCC) is an extremely rare finding and accounts for only approximately 0.5%–1% of all malignant pancreatic cancers (2, 3). Because of this infrequency, there are no established or standardized guidelines to treat SCC of the pancreas, and many different treatment regimens have been tried among the published case reports of pancreatic SCC. Here, we report a case of a 65-year-old Hispanic man who was diagnosed with pancreatic carcinoma with dominant squamous cell differentiation who achieved complete pathologic response (CPR) after treatment with gemcitabine, cisplatin, and nab-paclitaxel every 14 days for six cycles (**Figure 1**). This case is unique in that it is the first known CPR of a squamous cell pancreatic carcinoma published in the literature.

## Case report

A 65-year-old man with a past medical history of type 2 diabetes on insulin and hyperlipidemia on atorvastatin presented to the emergency department with complaints of umbilical abdominal pain radiating to his back for a year prior. He had experienced constant, increasing pain over the past month that resulted in difficulty in sleeping due to overwhelming pain. He had some itching of his palms bilaterally and associated fatigue. He denied any fevers, chills, night sweats, headaches, yellowing of his eyes or skin, nausea, vomiting, diarrhea, or unintended weight loss. He also was able to eat a significant amount of food and denied early satiety. He took no medications. He had no smoking history and drank 2 beers a month. He had no family history of pancreatic or gastrointestinal cancers. His physical exam was notable for epigastric pain to light palpation without visible nodules or masses. He had no jaundice or scleral icterus.

**Figure 2:**
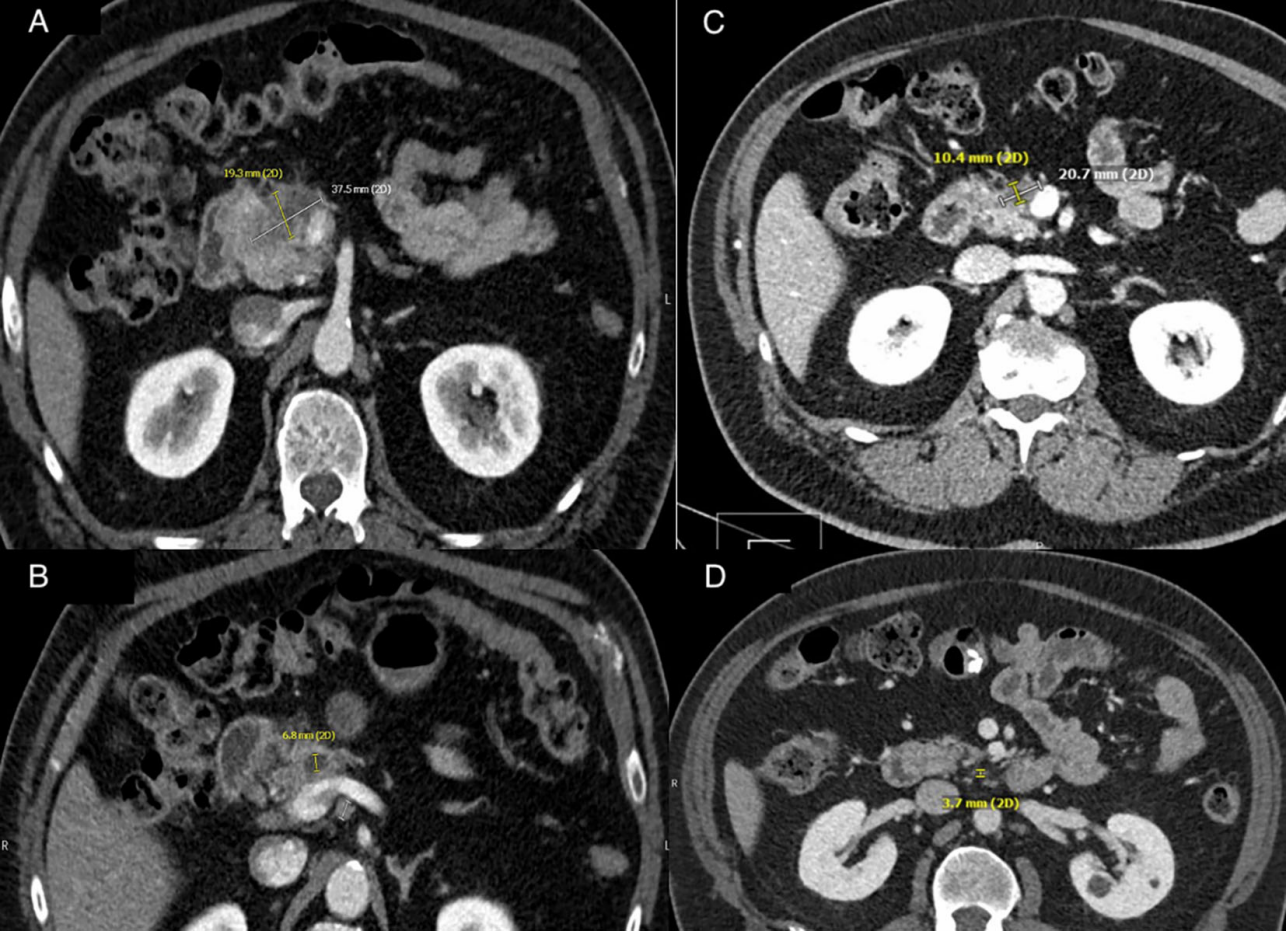
**(A)** CT abdominal imaging of pancreatic mass at time of diagnosis. A heterogeneous hypodense mass at anterior pancreatic head measuring 3.8 cm × 1.9 cm with associated pancreatic ductal obstruction. **(B)** CT abdominal imaging of lymphadenopathy at time of diagnosis. Lymph node posterior to the uncinate process measuring 10 mm on axial imaging. **(C)** CT abdominal imaging of pancreatic mass after five cycles of chemotherapy. Pancreatic mass with interval decrease in size to 2.1 cm × 1.0 cm. **(D)** CT abdominal imaging of lymphadenopathy after five cycles of chemotherapy. Interval decrease in size of lymph node posterior to the uncinate process measuring 3.7 mm on axial imaging. Created with BioRender.com.

His laboratory work was notable for a total bilirubin level of 0.3 mg/dL (reference, 0.4–2.0), aspartate aminotransferase of 18 U/L (reference, 15–37), alanine aminotransferase of 20 U/L (reference, 16–61), and alkaline phosphatase of 71 U/L (reference, 45–117). Computed tomography (CT) of the chest, abdomen, and pelvis was notable for a mass of 3.8 cm × 1.9 cm at the head of the pancreas concerning for a neoplasm with adjacent lymph nodes. Both the pancreatic mass and the lymph node were larger compared with that in an initial CT performed about 4 weeks earlier. The lungs were without metastatic foci (**Figures 2A, B**).

**Figure 3:**
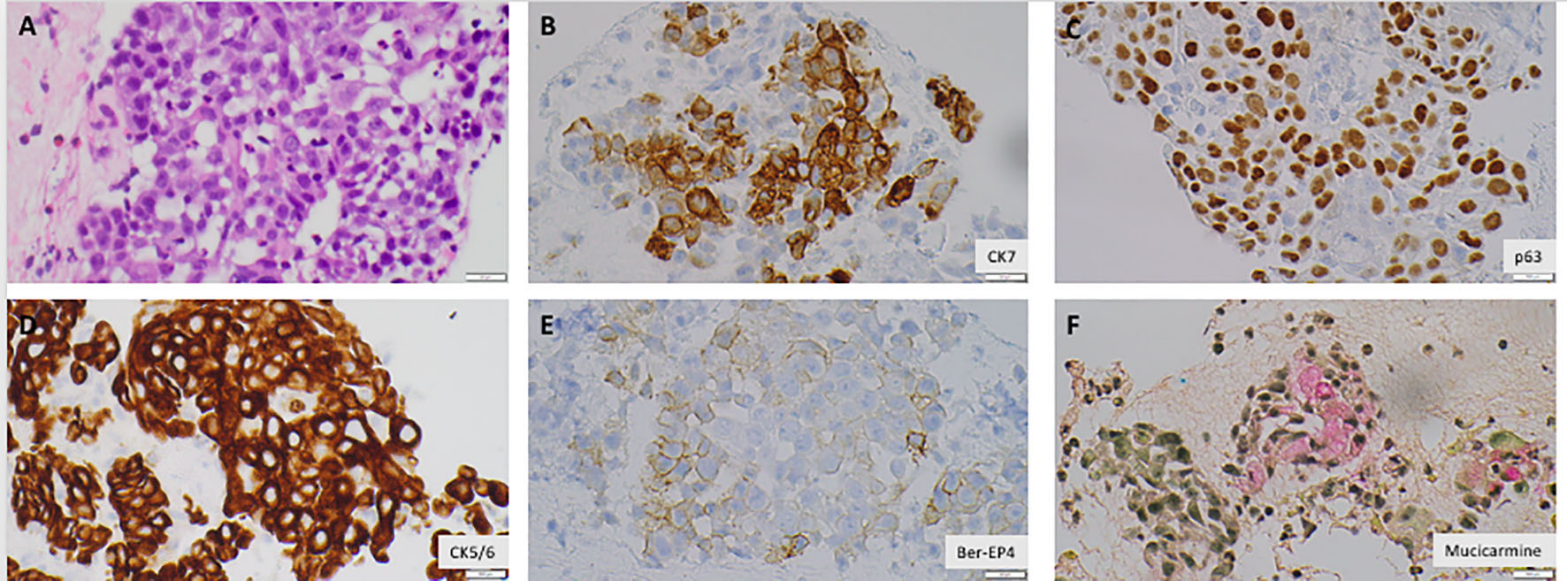
Malignant cells are positive for Cytokeratin 7, Cytokeratin 5/6, Epithelial Specific Antigen 4 (Ber-EP4) **(B, D, E)**, consistent with carcinomatous origin. The cells are also positive for p63, a squamous cell marker, mucicarmine, an adenocarcinoma marker, consistent with poorly differentiated carcinoma with squamous and glandular differentiations **(A, C, F)**. All images were obtained with a magnification of ×400. Created with BioRender.com.

He was referred to a tertiary care center for a biopsy and further care. A week later, he underwent an endoscopic ultrasound sonography (EUS) that showed a malignant-appearing pancreatic mass, 31 mm × 23 mm, at the uncinate process with upstream dilatation of the pancreatic duct and distal obstruction from the mass without dilation. There were no endosonographic abnormalities of the esophagus, stomach, and duodenum. The EUS-guided fine needle aspiration (FNA) demonstrated poorly differentiated malignant cells with both squamous and glandular differentiation (**Figure 3**). Direct communication with the pathologist rendering the diagnosis indicated that the squamous cell was the dominant feature. He had baseline tumor markers with a carcinoid embryonic antigen (CEA) of 2.4 ng/mL (reference, 0.0–3.0) and a carbohydrate antigen (CA) 19-9 of 93 U/mL (reference, <35). Next-generation sequencing of his tumor was notable for microsatellite stability, a programmed death ligand–1 percentage of 5%, with wild-type Neurotrophic tropomyosin receptor kinase 1/2/3/4, v-Raf murine sarcoma viral oncogene homolog B (BRAF), and Neuregulin 1 status. There was an insufficient quantity of malignant cells for whole-exome sequencing. We subsequently performed a positron electron tomography (PET)/CT that demonstrated a large f-fluorodeoxyglucose (FDG)–avid pancreatic mass compatible with the primary neoplasm and multiple avid lymph nodes consistent with nodal disease but no distant metastases.

Given the uniqueness of the tumor, the case was presented at a multidisciplinary tumor board meeting that recommended a combination regimen of gemcitabine (800 mg/m ^(2)^), cisplatin (25 mg/m ^(2)^), and nab-paclitaxel (100 mg/m ^(2)^) every 14 days for six cycles. We planned to refer for surgical intervention if subsequent imaging confirmed improvement.

The first cycle of chemotherapy was administered 3 weeks after his FNA. After completing four cycles of chemotherapy, he reported a complete resolution of his abdominal pain. His CEA remained within normal range at 2.8 ng/mL, and his CA 19-9 decreased to normal range at 19 U/mL. After the fifth cycle of chemotherapy, we performed a response evaluation CT of the chest, abdomen, and pelvis, which demonstrated a decreased pancreatic head mass size (2.1 cm × 1.0 cm), decreased lymph node size, and no new lymphadenopathy or metastatic disease (**Figures 2C, D**). He completed his sixth cycle of chemotherapy, and, 2 weeks later, he underwent a pylorus-preserving Whipple procedure with a hilar lymphadenectomy and falciform ligament patch on the stump of the gastroduodenal artery with negative intraoperative frozen section margins. The pathology showed no residual carcinoma with resection margins free of malignancy or high-grade dysplasia, with no malignant cells identified in all 20 lymph nodes. He was staged as an ypT0N0 (0/20)Mx after surgery. At 3 months follow-up visit with repeat imaging, CT chest, abdomen, and pelvis, no evidence for residual or metastatic disease was demonstrated. He recovered well from surgery and continues to live with high quality of life.

## Discussion

SCC of the pancreas is uncommon, as adenocarcinoma is the dominant histology. There are several theories speculating the etiology of primary SCC development within the pancreas. One theory suggests that primitive cells can differentiate into either a squamous or glandular histology (4). Alternative theories propose that a pre-existing adenocarcinoma can undergo squamous differentiation or that squamous cells in the ductal epithelium are capable of malignant transformation (4). In our case, the pathology report demonstrated both squamous and glandular differentiation, leading us to hypothesize that our patient had adenocarcinoma with squamous transformation resulting in mixed histology.

Thorough evaluation of a primary pancreatic lesion with squamous cell differentiation requires appropriate radiographic imaging of the thorax, abdomen, and pelvis as well as endoscopic evaluation to rule out a primary lesion sourced from another location, as this drastically impacts the treatment plan (5). Given that several previous case reports utilized PET/CT scanning for excluding other potential primary locations and for staging of pancreatic SCC, we chose to utilize the same imaging modality for our patient and advocate for the utilization of PET/CT for these purposes (6).

Given the rarity of pancreatic SCC, there are no formal, standardized guidelines on the treatment of squamous cell pancreatic cancer. However, there are several case reports that have proposed various potential treatment options. Ntanasis-Stathopoulos et al. performed a systematic review and pooled survival analysis of SCC of the pancreas that consisted of 54 cases with a mean age of 61.9 years (7). It was found that the median overall survival (OS) was 7 months, utilizing a variety of treatment modalities such as chemotherapy, immunotherapy, and surgical resection (7). The authors found that ability to be resected and more recent publication year were statistically significant when being associated with better OS, as was low to intermediate tumor grade. The authors suggested that further collaborative studies are necessary to validate the results found.

We performed an updated narrative review of case reports published after the 2017 review by Ntanasis-Stathopoulos et al. We performed a literature review of the PubMed database by using the search terms “pancreatic,” “squamous cell carcinoma,” “treatment,” and “primary” (**Table 1**). Two cases were excluded because the patients did not receive treatment. Articles that are not in the English language were excluded.

**Table 1:** Summary of case reports of primary squamous cell carcinoma published between 2018 and 2023.

Author	Age/Gender	Tumor Location	Treatment	Response
**Farokhi et al.** (8)	**57/M**	**Pancreatic head and liver metastasis**	**Leucovorin, fluorouracil, and irinotecan hydrochloride**	**1 month progression-free disease**
**Majumdar et al.** (9)	**60/M**	**Pancreatic tail**	**Neoadjuvant Gemcitabine and nanosomal paclitaxel lipid suspension with a pancreatico-splenectomy**	**2 months progression-free disease**
**Li et al.** (10)	**70/F**	**Pancreatic body**	**Laproscopic distal pancreatectomy and splenectomy**	**6 months progression-free disease**
**Alajlan et al.** (11)	**79/M**	**Pancreatic tail with invasion into splenic hilum and stomach body**	**“ palliative chemotherapy”**	**3 months overall survival**
**Yang et al.** (12)	**54/F**	**Pancreatic neck**	**Sintilimab and Gemcitabine**	**4 months progression-free survival**
**Zhang et al.** (6)	**64/F**	**Pancreatic tail with LN metastasis**	**Intensity-modulated radiation therapy with paclitaxel, cisplatin, and gemcitabine**	**8 months overall survival**
**Huang et al.** (13)	**52/F**	**Pancreatic tail with hepatic and LN metastases**	**Albumin-bound paclitaxel and cisplatin**	**13 months progression-free disease**
**Grant et al.**	**65/M**	**Pancreatic head with LN metastases**	**Gemcitabine, cisplatin, and nab-paclitaxel**	**Complete pathologic response without recurrence**

In our review of recent literature, the average age ranged from 52 to 79 with a mean age of 62.2 years. Similar to the review by Ntanasis-Stathopoulos et al., we found no preference in gender when comparing the cases. The treatments varied from combined immunotherapy, chemotherapy, and/or surgical intervention. Three of the seven case reports had disease localized in the pancreas. In our narrative analysis of the recent cases since 2017, we found the progression-free survival to be denoted at 5.28 months. From the findings of our narrative review and the review by Ntanasis-Stathopoulos et al., our case is unique in that, to our knowledge, it is the only case of pancreatic SCC to achieve CPR utilizing only chemotherapy as a treatment modality. Our regimen was similar to that of Zhang et al., who documented a pancreatic lesion with lymph node metastasis treated with intensity-modulated radiation therapy with four cycles of paclitaxel, cisplatin, and gemcitabine (6). We determined this regimen on the phase II clinical trial by Shroff et al., in which patients received gemcitabine (800 mg/m ^(2)^), cisplatin (25 mg/m ^(2)^), and nab-paclitaxel (100 mg/m ^(2)^), as they reduced the dosages due to hematological toxicities (8). In contrast to that in the work of Zhang et al., our patient was treated with an extended six-cycle course of treatment with chemotherapy, and we decided not to add radiation as part of pre-surgery treatment based on A021501 phase 2 randomized clinical trial showing no favorable benefit in OS in patients adding hypofractionated radiotherapy to FOLFIRINOX (9).

Immune checkpoint inhibitors have shown a great promise in a variety of SCC cancers (10–12). In particular, Yang et al. opted to treat SCC of the pancreas with 90% Programmed death-ligand 1–positive tumor cells using sintilimab (anti–PD-1) and gemcitabine, yielding a partial response (13). Because our patient had a PD-L1 percentage of only 5% and a lack of beneficial data for the use of check point inhibitor(s) in pancreatic adenocarcinoma, we decided against a combination of systemic chemotherapy with a check point inhibitor for our patient. Further research is needed to determine what the potential benefit immunotherapy could have in pancreatic cancer with squamous cell differentiation.

Huang et al. published a case report of a patient with stage IV pancreatic SCC with somatic Breast cancer 2 genes mutation, treated with cisplatin and nanoparticle albumin-bound paclitaxel (14). Similarly, the phase 1b/2 pilot clinical trial by Jameson et al. shows a high overall response rate in patients with advanced pancreatic cancer treated with a triple regimen of cisplatin and albumin-bound paclitaxel plus gemcitabine (15). Therefore, although the initial regimen planned for our patient was FOLFIRINOX, the regimen was changed to cisplatin, nab-paclitaxel, and gemcitabine after the pathology report for our patient showed dominant squamous cell differentiation. This was due to the reason that irinotecan did not show satisfying benefit in SCCs in general, such as in the study by Bai et al. who evaluated liposomal irinotecan and 5-fluorouracil in SCC of the esophagus and head/neck (16).

This case report is the first known case of a primary squamous cell pancreatic carcinoma utilizing systemic therapy to achieve a complete pathological remission. This case sheds light on potential duration and type of antineoplastic treatment needed to adequately treat pancreatic carcinoma with squamous cell differentiation. However, there are limitations in that this is a case report of a singular patient, and larger cohort studies are needed to further define the treatment algorithm for pancreatic SCC. This case and its accompanying narrative review highlights a need for guideline-directed treatment regimens to help improve the prognosis of patients with pancreatic SCC. There is a further need to determine the ideal treatment regimen for SCC of the pancreas.

## Data availability statement

The original contributions presented in the study are included in the article/supplementary material. Further inquiries can be directed to the corresponding author.

## Ethics statement

Written informed consent was obtained from the individual(s) for the publication of any potentially identifiable images or data included in this article.

## Author contributions

CG: designed the study, provided clinical review, provided literature review, and drafted and reviewed all versions of manuscript. ZA: provided data interpretation, editing, oversight, and reviewed all versions of the manuscript. F-CL: conceptualized the study, provided direct patient care, data interpretation, editing, oversight, and reviewed all versions of the manuscript. TT: provided pathologic figures, data interpretation, editing, and reviewed all versions of the manuscript.
